# RNA Sociology: Group Behavioral Motifs of RNA Consortia

**DOI:** 10.3390/life4040800

**Published:** 2014-11-24

**Authors:** Guenther Witzany

**Affiliations:** Telos-Philosophische Praxis, Vogelsangstraße 18c, 5111-Buermoos, Austria; E-Mail: Witzany@sbg.at; Tel.: +43-6274-6805; Fax: +43-6274-6805

**Keywords:** natural codes, some -somes, biotic split, virosphere, competent *de novo* generation

## Abstract

RNA sociology investigates the behavioral motifs of RNA consortia from the social science perspective. Besides the self-folding of RNAs into single stem loop structures, group building of such stem loops results in a variety of essential agents that are highly active in regulatory processes in cellular and non-cellular life. RNA stem loop self-folding and group building do not depend solely on sequence syntax; more important are their contextual (functional) needs. Also, evolutionary processes seem to occur through RNA stem loop consortia that may act as a complement. This means the whole entity functions only if all participating parts are coordinated, although the complementary building parts originally evolved for different functions. If complementary groups, such as rRNAs and tRNAs, are placed together in selective pressure contexts, new evolutionary features may emerge. Evolution initiated by competent agents in natural genome editing clearly contrasts with statistical error replication narratives.

## 1. Introduction

In contrast with the evolutionary narratives favoring selection of random replication errors of nucleic acids that constitute the genetic text, we do not know any real-life languages or codes that have emerged as randomly derived replication errors of sequences of characters [1,2]. Every natural language is based on signs, whether they are signals or symbols. In humans and other animals they are transported auditively, visually or tactilely. In non-human living beings, such as plants, fungi and prokaryotes they are transported by small molecules in crystallized, fluid or gaseous forms [3,4,5,6]. Additionally these signs are combined coherently with combinatorial rules (syntax) to transport increased levels of content. Signs are not generated and used by themselves, but by living agents. These sign-generating and sign-using agents live in continued changing interactions and environmental circumstances. This is the real-life context (pragmatics) in which all living agents are interwoven. Most importantly, context, not syntax, determines the meaning (semantics) of the signs in messages that are used to communicate and to coordinate group behavior. This is precisely why the same sentence, or the same syntactic sequence structure of any language or code, can have different and in extreme cases opposite meanings and therefore can be represented by different functions [7]. In consequence we can look at the genetic storage medium DNA as an ecosphere habitat and its natural inhabitants of abundant RNAs that edit the genetic code [8,9,10,11].

The coordination of the social behavior of living agents follows rules that are additional to natural laws. This is the reason why natural sciences, such as physics and chemistry, cannot explain biological communication, because the rule-following of living agents that communicate is different to underlying unchangeable natural laws [12].

## 2. RNA Structural Agents

### 2.1. Important Riboagents Act as RNA Stem Loop Groups

If we look at behavioral motifs of RNAs in addition to their physical chemical reactions, we focus on an additional level of empirical investigations. Groups (consortia) and their single members can change their behavior according to adaptational and coordinative needs—this means there are certain categories of group behavior. One of the most basic group behaviors is the self/non-self differentiation competence, which implies activities that maintain group membership and in parallel with the rejection/defense of non-group members. Only if group identity can be reached, it is possible to generate and use commonly shared rules to correctly combine signs to sign-sequences according to contextual needs. Group behavior in this perspective is completely missing in abiotic nature, no syntactic, pragmatic and semantic rules of sign-use are present.

If we look at the sequence structure of nucleic acid codes, some RNA stem loop groups are currently known to play vital roles in editing genetic text as a read and write medium [13,14]. This means their sequence-specific identification competence for insertion and deletion activities, which alter semantic content (the function that leads to altered regulation or altered protein production) of primary transcripts out of DNA [15]. Although all RNA secondary and tertiary structures can be objects of sociological investigations, in RNA groups the hairpin motif represents the basic motif.

Some ribonucleoproteins (RNPs) such as editosomes, spliceosomes and ribosomes are constituted of a variety of RNA stem loops that counter-regulate themselves during assembly in various steps and substeps. Also, small changes in the composition structure destroy their functionality. It must be assumed that the assembly occurs complementarily,* i.e.*, although different parts were derived originally for different functions, now all parts play an important role in coordinated interaction and the consortium must be established as a whole to function appropriately [16].

**Figure 1:**
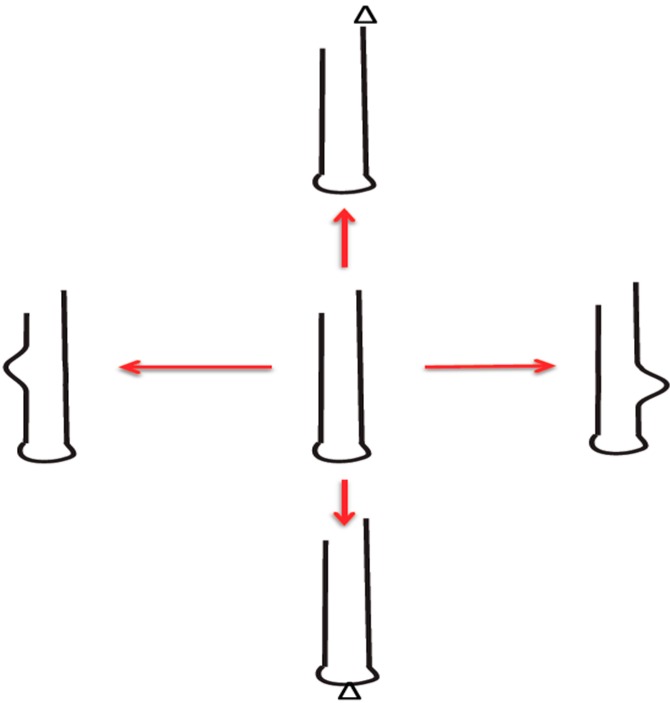
Self-generating RNA-stem-loop innovations: Generation of diversity (innovation) is shown. This is different from errors (accident, damage). Red arrow denotes diverse products from central template by low fidelity replication. Bulges and triangles denote changes. (Figure and text from reference [19]).

The RNA stem loop or “hairpin” loop is the basic motif with which RNA agents combine to form the more complex agents we know as tRNAs, rRNAs and self-splicing group II introns [17,18,19]. Every single RNA molecule is part of such a stem loop structure (Figure 1). When we consider early and recent RNA world agents, we think of the stem loop structures that build groups that essentially act on all relevant processes of life [20,21].

A crucial competence for group building is RNA ligation [22,23]. Without ligation only single RNA stem loops of rather limited size would exist and could not form complex secondary structures with their complex functions. The ligation of multiple RNA stem loops into a functional ribozyme constitutes group identity, which is different from others that do not share this identity. Such RNA stem-loops may derive from nucleases that “recognize” (identify) and cleave specific nucleic acid sequences (additionally to their competence in several DNA repair pathways) and therefore serve for reusable RNA stem loop tools. This complementary function of nucleases and ligases on the RNA stem loop level possibly initiates the biological functions of group identity building and self/non-self identification competences. Together they represent essential tools in other “addiction”-modules (see below) such as restriction-modification.

### 2.2. Addiction Modules: Successful Cooperative Motif for Invading DNA Habitats

RNAs that can form both simple base-pairing stems with non base-pairing loops by self folding, as RNA stem loops become subject to biological selection. RNA groups representing information and catalytic function provide themselves with a biotic feature such as self/non-self differentiation competence, acting together in variable contexts and with clear behavioral motifs, such as the natural genome editing of themselves, as well as of the DNA storage medium of their cellular host organisms. Now, we must ask how do RNA stem loop groups achieve the intriguing capability to colonize the DNA of host organisms? How do they organize themselves into being appropriate modules for reuse in DNA habitats by their cellular host or into being active (or silenced) as a regulatory element for gene expression?

One of the most important behavioral motifs is termed ‘addiction module’, linking originally competing functions: the former competitive groups are now linked together in a counterbalancing manner, serving as the identity feature within a host genome. This implies that disturbances within this counterbalanced module can result in massive deregulation of genetic functions that we can identify in a variety of diseases.

Generally, this behavioral motif of complementarity building without destroying one of the participating modules to create a unique structure that functions as an identity feature for the genotype of a host represents a key behavior in evolution: it transfers multiple genes within one infection event and contains multiple parts in parallel. In this respect I would like to predict that the more regulation features in living organisms we can look at, the more such addiction modules will be detected.

Addiction modules can be defined as features that consist in general of a stable toxic component that is counterbalanced by an unstable component, which inhibits and suppresses the toxic component [24,25,26]. Both are necessary for transferring a feature to the host without harming the host. In the case of a restriction/modification module this means that, for example, 52 restriction enzymes are counterbalanced by 52 modification enzymes. This indicates how complex addiction modules are constructed and how difficult it can be to understand the evolution of such phenotypes [27,28].

Several kinds of addiction modules are known. First, and most prominent in bacterial life, is the restriction/modification addiction module, which is a common feature with immune functions. One part consists of an antitoxic modification enzyme, which is an unstable beneficial (protective) agent. The counterpart consists of a toxic restriction enzyme component, which is a stable but harmful (destructive) agent. Another kind of addiction module consists of two related features. There is an antitoxic antipore toxin that represents the unstable protective agent and a toxic component with a toxic pore, which represents the stable but destructive agent [29]. A third kind of addiction module consists of the antitoxic viral immunity component and the toxic component of viral-mediated lysis [30]. This third kind is the most obvious viral-derived immune function, because it necessarily consists of a persistent genetic parasite and an external lytic phage [31].

Additionally the basic motif of a counter-regulated module reminds us on structures with similar features, such as the nuclease/ligase regulation (see above), as basic competence for self/non-self differentiation within RNA stem loop groups [32,33,34], the kinein/dynein retroviral movement pathway [35] or the viral origin of some ribonucleoproteins [36]. Also the telomere/telomerase dependent genome maintenance [37] and the function of centrosomes and spindles [38,39] most seemingly represent counter-regulated addiction modules, although their origin still lasts in the dark.

If we look at the virosphere, we can find some basic motifs of group building and group cooperation between genetic parasites that act in a similar way and are cooperatively supported by subviral agents, defectives, or remaining viral parts that can be reused in other contexts. We can find them as genetic parasites, mixed parasites (epiparasites), hyperparasites (parasites of parasites), cooperating or competing according to changing circumstances in various ways [31].

### 2.3. Evolution of New Motifs through Kissing and Pseudoknotting

Key to generating the RNA structural motifs is the ability of a RNA to fold back to itself and build groups (consortia) of such stem loops via ligase procedure out of nuclease generated modules. The folding loop remains a single-stranded RNA sequence. Various motifs have been identified, yet all of them share a common function: they stabilize RNA tertiary formation. Such motifs include pseudoknots, kissing loops, A-minor motifs, A-platforms, kink-turns, S-turns, tetraloops and their receptors, and a variety of non-canonical base-pairs and base-triples [40].

Consortia of single RNA sequence strings build one of the most interesting structures: The pseudoknot is composed of two helical segments connected by single-stranded regions or loops that base-pair with complementary nucleotides [41]. Bases in the single-stranded loop are base-pairing with bases outside the loop elsewhere in the RNA sequence. The results of these interactions may lead to the catalytic core of key players of the present RNA world,* i.e.*, ribozymes, self-splicing introns, telomerase and ribosomal frameshifting in several viruses.

Interestingly, the base-pairing in pseudoknots is strictly context-sensitive and base-pairs overlap with one another in sequence positions. This imposes limits on algorithm-based prediction models, such as dynamic programming or stochastic context-free grammars [41].

Sequence specificity is not required for the various functions, but forms the structure, that is, a tool for appropriate pseudoknot function. The building-up of ribosomal subunits, ribozymes, and reverse transcriptase, such as telomerase, depends on pseudoknots. Replication, as well as RNA processing and inactivation of toxins, gene expression control are additional functions. Last but not least, pseudoknots are involved in translation modification such as internal translation initiation, ribosome rescue and frameshifting [42]. This indicates important roles in the evolution of the protein synthesis also.

The most important role of pseudoknots seems to be genetic control and feedback via cooperative motifs of pseudoknots with riboswitches in destabilizing ligand-free or stabilizing ligand-bound conformations [43,44]. Pseudoknots also offer binding sites for proteins or single-stranded loops of RNA. Pseudoknotting can be viewed as the most efficient way of folding RNAs in an active conformation. Long-range interactions are also possible for organizing global folding and linking separate domains of RNA together. This represents an essential competence for *de novo* generation of sequence space. Pseudoknotting seems to be a special viral competence, including the control of virus genome translation, replication, the switch between translation and replication, and genome packaging, later transferred to cellular domains via persistent infections.

A special RNA folding motif of the class of pseudoknotting is represented by stem-loop “kissing,” in that single-stranded regions of RNA stem loops bind according to molecular syntax rules to other single-stranded stem loop structures to unite and build more complex group identities [45]. In kissing loops, the single-stranded sequences of RNA stem loops initiate the search of homologs within the complementary sequence syntax of other stem loops [46]. Interestingly, those replicating RNA agents that can modify their sequences to increase stem loop formations, which can act as kissing modules, will replicate faster and more efficiently than competitors [47]. The self/non-self differentiation clearly depends on competence for interaction based on molecular syntax. Additionally, it looks likely that stems arrange the proper positioning of the loops to base-pair with others [48].

## 3. Basic Modules for RNA Group Building

For several decades the mainstream perspective on non-coding RNAs resulted in the assumption that they are useless remnants of former evolutionary stages, which represent “junk”. This perspective is now falsified. Non-coding RNAs play vital roles and function in gene regulation and coordinate, as well as organize various actions, such as chromatin modification, epigenetic memory as a prerequisite of learning processes, transcriptional regulation, control of alternative splicing, RNA modification, RNA editing, control of mRNA turnover, control of translation and signal transduction [49].

Most of these non-coding RNAs are divided into smaller RNAs that are integral parts of ribonucleoprotein (RNP) complexes. Small RNA species include micro RNAs, small interfering RNAs, small nuclear RNAs and small nucleolar and transfer RNAs [50]. Although recent research has tried to evaluate the enormous regulatory networks of small RNAs, the role of thousands of longer transcripts was not so clear [51]. Additionally they play important roles in histone modification and methylation, that is, epigenetic control of developmental processes [52], and also transcriptional interference, promoter inactivation and effects on enzymatic pathways. Interestingly, these large non-coding RNAs are found as interlacing and overlapping sense and antisense transcripts derived from introns or intergenic regions. Similarly to their smaller relatives, they are involved in the formation of RNP complexes.

Micro RNAs and their associated proteins are ribonucleoproteins. Small non-coding RNAs also share a special competence regarding epigenetic regulation of gene expression and are derived from repetitive genomic sequences [53]. The capacity for epigenetic regulation of gene expression includes the “recognition” (identification) of specific sequences in other nucleic acids and is common to RNAs [54], especially small nuclear RNAs and tRNAs that identify splice junctions in both pre-mRNAs and codons, and process both the subunits of the spliceosome and the ribosome (see below).

Interestingly, small nuclear RNAs and small nucleolar RNAs counter-regulate activities which indicate similarity to an “addiction” module. Small nucleolar RNAs guide pseudouridylation and 2’O-ribose methylation of rRNAs in forming duplexes with their target. Small Cajal body-specific RNAs guide modifications of splicesomal RNAs. Small nucleolar RNAs have been identified as a family of mobile genetic elements [55] and other small RNAs derived from snoRNAs [56], which suggests that they are the most ancient and numerous family of non-coding RNAs [57].

### Two of a Perfect Pair: Micro RNAs and siRNAs

Small RNAs such as endogenous miRNAs and siRNAs share biogenesis and can perform interchangeable functions [58]. They cannot be distinguished by their chemical composition or their action, but they differ in their production pathways: microRNAs derive from genetic sequences that are different from known genes, whereas siRNAs derive from mRNAs, transposons and viruses. MicroRNAs are processed out of transcripts that can form RNA stem loops, whereas siRNAs are processed from long RNA duplexes. MicroRNAs are always conserved in related organisms, whereas endogenous siRNAs are rarely conserved. MicroRNAs are produced from genes that are specialists in the silencing of different genes, whereas siRNAs are typically auto-silencing, such as viruses, transposons and repeats of centromeres [59,60].

SiRNAs are expressed by extended double-stranded regions of long inverted repeats, can inhibit the expression of nearly any target gene in response to double-stranded RNA and have a very efficient and ancient immune function against genetic parasites [61,62]. They function by identifying foreign RNA sequences and inhibiting their replication. The ancient RNAi immune function is based on this self/non-self identification competence [61,63]. Many of these elements are retroposons or transposons and are encoded in the repetitive sequences of the genome [62].

Small interfering RNAs or microRNAs act in a coordinated manner in that they share the division of labor in hierarchical steps of suppression and amplification. This is indicated in transposable elements that encode both siRNAs and miRNAs [64]. They can be found in intronic regions and build stem loop structures as a common feature of active RNA species.

The defense mechanism of host genomes against transposable element invaders through siRNA evolved into miRNAs with a new regulatory complexity and a new phenotype. First evolving as an immune function, they were later co-opted as a tool for complex regulatory pathways in host gene expression [64]. This exapted co-option of identical competencies for different purposes seems to be a common evolutionary pattern for regulatory controls that can be flexibly altered and rearranged to cause phenotypic variation without altering basic components [65].

## 4. The Biotic Split: From Molecular Random Assemblies to RNA Group Behavior

If we look at the crucial difference between the physical chemical properties of RNAs and their social group behavior, we see that single RNA hairpins act exclusively according to physical and chemical laws, which do not underlie natural selection processes. In stark contrast, groups (consortia) of RNA stem loops generate behavior that underlies biological selection [20,21,22]. Coherent with the essential features of natural languages and codes the nucleic acid code as a biological code functions only in social interactions or more precisely, natural code use is a kind of social interaction [12,66]. This means single exemplars alone would not function as a natural code but only as molecules that react in physical chemical reactions. Only if many such elements come together they act in a biological fashion as real code,* i.e.*, additionally to physical and chemical laws they coordinate group behavior in accordance with pragmatic, semantic and syntactic rules.

Simple RNA structures can become biologically functional if they are used and integrated within higher order structures. This means single RNA nucleotides are not functional unless they become part of group identities. To encode content (information), social interactions (function) by duplex building and further ligations are necessary to form identity groups. This parallel set of levels—information and function—designates the fundamental split between inanimate and animate nature that can be found here, the absence or presence of social interactions based on natural codes.

**Figure 2:**
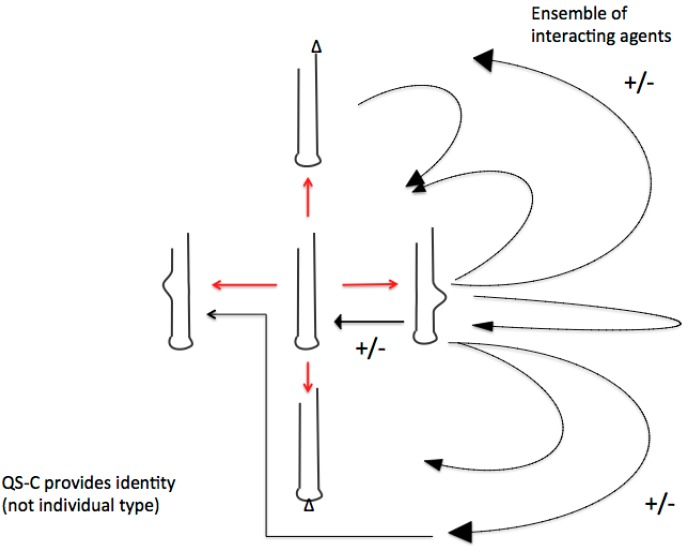
The crucial difference of quasi-species consortia with former quasi-species-concepts (fittest type—mutant spectra) is the basic consortial organization of functional RNA ensembles. Shown below are the possible consortial interactions (black arrows) of just one the diversified RNA-stem-loops. These multiple activities (shown as +/−) preclude individual fitness definitions but require emergence and adaptation of group membership-identities. Defectives with similar subviral RNA-(stem-loop groups) remain relevant in both evolutionary and developmental processes. As a result of this basic evolutionary process of RNA stem-loop consortia building we can look at the emergence of *de novo* identities. (Figure and text from reference [19]).

This was precisely the proposition of Manfred Eigen when he introduced his quasispecies and hypercycle concept (“... behavior of coupled cycles, the properties of which resemble, in many ways, social behavior”) [67]. Unfortunately, Eigen did not mention the additional level to physical chemical reactions,* i.e.*, rule-governed interactions. The RNA sociology perspective therefore contrasts the narrative of the genetic code as a molecular random assembly, as being obviously the result of competent editing by social interacting RNA groups (Figure 2).

### 4.1. The Editosome Competence

Let us take a look at some such code-editing RNA groups and the essential ribonucleoproteins that act on the nucleic acid language. They must share a group identity, which is able to reject/defense non-group members. Additionally they must identify content (information),* i.e.*, the correct site of the nucleic acid sequence to edit nucleotides. We can speculate that they identify sequence syntax or specific markings on the sign sequences that are relevant to common interactions that are subject to change. Since the rise of epigenetics, the marking of sequences is an important designation of how to identify sequence syntax for modification. The evolutionary derivation of epigenetic markings is not answered so far, although agents of the virosphere are known to be most relevant in this competence.

We can start with an important context-dependent sequence operator, the editosome. RNA editing is a co- or post-transcriptional process that alters the RNA sequence derived complementarily to the DNA from which it was transcribed. RNA editing changes gene sequences at the RNA level. The edited mRNA specifies an amino acid sequence that is different from the protein that could be expected and is encoded by the genomic DNA of the primary transcript [68,69]. RNA editing alterations of such transcribed RNA sequences occur by modification, substitution and insertion/deletion processes [70]. Sequence alteration that changes the genetic meaning of a transcript is termed editing, whereas structural changes are called modifications [71]. tRNA editing shows some features of quality control, especially the precise recognition of substrates and substrate discrimination capability in a pool of nearly identical substrates [72]. The end-products, such as RNAs or proteins, can be different, although the encoded DNA gene remains the same.

RNA editing must “recognize” (identify) the site that has to be edited. This is not a random process and needs some *cis*-acting signals. In a second step, the editing site has to be processed by RNAs or proteins. In a further step, enzymatic agents have to be directed to the editing site to process the editing. All steps are catalyzed by a ribonucleoprotein complex known as the editosome. Additionally, the editosome is constituted of a variety of subcomponents, which counterbalance each other [73]. RNA editing and RNA interference are in competition, which indicates also some sort of an “addiction” module [74].

### 4.2. The Spliceosome Competence

The variable meanings of identical sign sequence syntax are an inherent feature of natural languages and codes. This optimizes energy costs in that informational content fixed in an information storage medium can be modified according to environmental needs in different ways,* i.e.*, with more *than o*ne meaning (function). Additionally, the code modification can reach high levels of complex interactions, which multiply the possible outcomes. After the editosome another ribonucleoprotein complex acts on the result of the editosome: the spliceosome.

It is assembled in various steps and cuts out introns and splices exons together to form the final protein coding set [75]. RNA editing and splicing are heavily interconnected, so that the editosome and spliceosome are important co-players [76]. The spliceosome is a group of small nuclear RNAs interconnected with at least 300 different proteins.

Interestingly, the variety of steps in which the subunits of the final spliceosome are produced are counterbalanced within competing parts that regulate the stepwise processing of subcomplexes of the spliceosome. Besides the H complex, the E complex, the SR proteins within the E complex, the A complex, the B complex and the C complex are required steps before the final exon ligation processing occurs.

After this final splicing procedure of the mature spliceosome, the remaining RNA products are actively discharged from the spliceosome and recycled for further catalytic processes. Depending on the regulations, the end-product may vary concerning the context dependency of the regulation process, which is highly sensitive to various needs and circumstances. In consequence, spliceosomal regulation differentiates the inclusion or exclusion (enhancers or silencers) of exons in the final mRNA [77]. Splicing regulation occurs by means of competing *cis*-acting elements that precisely balance regulatory proteins. This means that the competition of exon ligation and exon rejection is instable from the beginning until the end of spliceosome assembly. As a consequence, alternative splicing is regulated until the latest stage of splicing actions.

The crucial competence of the spliceosome is the appropriate and precise splice site “recognition” (identification) of consensus sequences that define exon-intron boundaries [78,79]. Depending on the flexibility of splicing in the various time windows of spliceosomal assembly, the correct identification of sequence recognition is a multiple competence that may change quickly [80]. Interestingly, many spliced introns are precursors of other encoded RNAs, e.g. small nucleolar RNAs, microRNAs, and long non-coding RNAs, or they remain longer in the cytoplasm and play a role in translation steps [75]. Some introns like group II introns act as ribozymes themselves to splice RNA and invade DNA [81].

### 4.3. The Ribosome Competence

After the RNA groups of editosome and spliceosome have acted to arrange a coherent nucleic acid sequence and are ready for translation into proteins, a third RNA group becomes active: The ribosome is the key player in translation after RNA editing and RNA splicing. Because the ribosome is at least the most important player in cellular life replication and is absent in viral genomes, it must have been a crucial evolutionary step from the capsid-encoding organisms of the pre-cellular RNA world and the ribosome-encoding organisms in the cellular life world [82].

Transcription and assembly of the two ribosomal subunits occur in the nucleolus, which itself must be assembled by a highly regulated process during teleophase [83]. The nucleolus assembly is counter-regulated by phosphorylation/dephosphorylation during the transcription process and additionally, the RNP processing complexes under the control of the CDK1-cyclin B kinase and PP1 phosphatases [84].

Ribosomes are comprised of two-thirds RNA and one-third protein (by mass). As we understand it, ribosomal proteins are vital for structural stabilization. Around the catalytic site of the ribosome there are only RNAs and no ribosomal proteins [85]. This means that the ribosome is originally a ribozyme, and proteins are not involved in the catalytic activity [86,87]. Also the subunits of the ribosome emerged out of different RNA stem loop groups the evolved originally for different purposes [85,88].

### 4.4. Bypassing Translation Leads to New Amino Acid Identities

One of the outstanding explorations in the last decades was the multiple ways how identical nucleotide sequences that serve as genetic storage medium undergo a variety of modifications during transcription as outlined above. The translational process from DNA via RNA and an abundance of regulatory processes of non-coding RNAs is object to a further process to dynamically modify genetic storage semantics. During the translational process, ribosomes may bypass and therefore ignore parts of the mRNA sequence. In this behavioral motif, translation is blocked at a certain codon, and a different stem loop RNA ensures the further translation process at a certain stage of the mRNA [89]. In the mitochondrial genome of a yeast species, 81 translational bypassing elements were found recently. The mitochondrial bypassing resembles a sequence of the t4 phage gene 60 [90]. Interestingly, some codons remain unused in mitochondrial translation and can be used as variable module-like parts in new ways, such as serving as translational barriers for bypassing undesired sequences, or they may be reassigned to new amino acid identities by a codon capture procedure. As such bypassing elements resemble mobile genetic element dynamics detection of their evolutionary origin should be interesting [89,91].

### 4.5. The Secret Life of RNA Stem Loop Groups Which Build tRNAs

Alan Weiner and Nancy Maizels showed convincingly that transfer RNAs represent a combination of two formerly different modules [92,93]. The two parts did not evolve for protein synthesis. One half served to mark single stranded RNA for replication in the early RNA world. The other half of the tRNA is a later acquisition. In Nanoarchaeota [94], the various tRNA species are encoded as two half-genes, one encoding the conserved T-loops and 3’ acceptor stem, the other encoding the D-Stem and the 5’ acceptor stem subunit. In Nanoarchaeota, the CCA sequence which is important in tRNAs for protein synthesis in nearly all cellular life is not encoded in the tRNA genes but is added posttranscriptionally by an enzyme [95]. It seems that the evolution of protein synthesis has coordinated a variety of older genetic agents and represents another example of co-opted adaptation [96,97].

Pre-tRNAs perform self-cleavage, which is clearly a ribozymatic reaction independent of translation [98,99,100] and tRNAs may serve as precursors of small RNAs. tRNA fragments serve as signals for acetyl synthetase, apoptosis and others [101]. Fragments of tRNAs are also involved in the regulation of various processes including replication of plasmids. There is also evidence that tRNAs can flow back to the nucleus from their site of action in the cytoplasm [98].

## 5. Inheritable Conservation of New Experiences

The rise of epigenetics is more than an epiphenomenon within molecular biology. It marks an important perspective in which the given genetic information is marked according to different expression patterns depending on the tissue specificity and according to environmental conditions that make it necessary to change expression patterns [102,103]. One could see it as an environmental memory of the available genetic information according to adaptational needs: one genome out of which multiple cell types can be processed.

Chromatin marking represents a further behavioral motif that enables a kind of identity programming, which may or may not be heritable. This means a specific cell within an organism is able through epigenetics to obtain an identity or even to change its identity if environmental conditions make it necessary. Only RNAs are able to mark DNA sequences. RNA agents are mobile and can serve as signals throughout tissues, organs and whole organisms. In this respect the imprinting of new experiences that leads to variable meanings of genetic information depends on the ability of non-coding RNAs [104,105,106]. With epigenetic marking life has an appropriate tool for the emergence of memory and learning processes for better adaptation [107,108].

Both small RNAs and long non-coding RNAs are competent in terms of directing chromatin changes through histone modifications and DNA methylation. These non-coding RNAs are able to direct chromatin-modifying agents to specific targets. Interactions between non-coding RNAs and such agents are components of these regulatory networks [109]. In small RNA-driven silencing pathways, the regulatory RNAs identify and mark potentially dangerous “non-self” elements for transcriptional silencing or elimination [110]. In other networks homology between the regulatory RNA and the target locus marks the region as ‘‘self’’ and protects it from silencing or elimination.

As mentioned before the evolutionary derivation of this special competence to identify specific sites for editing, splicing or epigenetic markings is not answered, although agents of the virosphere are known to be most relevant in this competence. Therefore epigenetic markings too, which are common in the virosphere for fixing identity of agents and enabling the differentiation between self and non-self, seem to be exapted to the world of DNA-based cellular life.

## 6. Endogenization Experts and Persistent Symbiotic Lifestyle

Current knowledge about the virosphere and virolution indicate interactions of RNA viruses, DNA viruses, RNA-DNA viruses, viral swarms, viral and RNA-based sub-viral networks that cooperate and coordinate (regulate) within cellular genomes either as replication-relevant co-players or suppression-relevant silencers [111,112,113,114]. Some represent infection-derived modular tools of non-coding RNAs that have built consortia of complementary agents that function together [115].

Persistent viral lifestyles are counterbalanced by viral properties found as addiction modules, that transfer complete genetic data sets into host genomes and alter the DNA genetic identity of the host and, additionally, the formerly competing viral agents, without damage to the host genome content [116]. As we saw above, RNA consortia edit genetic code without altering inherited DNA content. Transcribed from DNA cellular sequences, the RNA-activated inhabitants from former viral infection events act as modular tools for cellular needs in nearly all-cellular processes [117]. That they act not as lytic agents but as part of the host genetic identity is the result of inhabitation by counterbalanced competing genetic parasites [116]. Addiction modules not only change genetic identity but also enrich immune functions of host organisms to fight related parasites. We can identify these in the known complex adaptive immune systems [24,27].

Endogenous retroviral competences in the persistent status are often characterized by features expressed only in the strict time window of a developmental process, such as axis formation, trophectoplast formation (as in the case of endogenous retroviruses in placental mammals), or the S phase of the cell cycle. In these highly specialized contexts, they are replicated through signaling, which blocks the suppression of the replication process. After the function is fulfilled, a signal once again initiates suppressor function. Retroelements—with their (1) higher-order regulatory functions, (2) ability for genetic creativity and (3) capacity for innovation of new regulatory patterns and combinations—descended from retroviruses, which can easily be identified by their three essential parts *gag*, *pol* and *env* [118]. Most endogenous retroviruses have been degraded into formerly connected domains, but they can still be recognized by retroposons or one of these three genes [119]. This means their formerly connected genomic content may be used by host organisms as single or networking modular tools for a variety of new regulatory functions [120].

The lifestyle of endogenous retroviruses and other endogenous viruses that compete or cooperate was long assumed to be a rare and exceptional phenomenon. Today we know that this is common use and DNA generally serves as habitat for RNA inhabitants in abundant numbers [15]. In most cases RNA viruses do not remain as functional agents but have lost several of their abilities [121,122]. Remaining parts like LTRs can be used by the host organism for other gene regulations. Mobile genetic elements are the remaining parts of such infectious agents [123]. It seems likely that in the virosphere we can find all the remnants of infectious RNAs that transfer RNA capabilities into the DNA world of host organisms.

## 7. Conclusions

We saw a variety of behavioral motifs of RNA groups that, although constituted by molecules, behave like living agents. They infect host DNA, settle within nucleic acid sequences, differentiate between self and non-self group membership, reject foreign members, assemble to cooperative groups that build essential tools in DNA based cellular life and identify relevant syntax in certain sequence sites, store environmental experiences into DNA content and act according circumstantial needs, to faster adapt to changing environmental situations. This means, additional to underlying natural laws, they follow rules that are absent in inanimate nature.

As in all known natural languages or codes, in the nucleic acid code we can also identify agents that generate and use code by an inherent competence to correctly combine signs to sign sequences according to contextual needs. These agents in most cases are infective RNAs, such as viruses, and sub-viral agents, such as viroids, and other related genetic parasites. Such competent agents interact, cooperate and integrate in host genomes where they are fully integrated or, in most cases, remain as defectives serving as module-like elements for co-opted adaptations in host gene regulation. As we now know, the RNA nucleic acid sequence is subject to a variety of alterations and modifications after or parallel with transcription out of DNA. Crucial for the function of such alterations is a precise site “recognition” (identification) where the change operators start or stop their competent action. This competence recalls phenomena such as self/non-self differentiation as a pre-requisite for memory and learning.

Whereas interactions between molecules and atoms underlie natural laws strictly, social behavior of agents in animate nature follows additional motifs, such as rules that govern sign-mediated interactions. In contrast to natural laws, such rules may change according to contextual circumstances to adapt faster to appropriate behavior and additionally are completely absent in inanimate nature. Living agents in populations that interact according to rule-governed sign-mediated interactions are therefore both able to coordinate behavior and able to install *de novo* generation of behavioral patterns as well as *de novo* generation of new sequence content. Competent *de novo* generation of new sequence content is completely different from statistical error replication. This was the finding of a decade-long discussion on the essential features of natural languages or codes.

The challenge is to think about agents that are able to generate nucleic acid sequences *de novo* in terms of social sciences (consortia, cooperatives, coordinated behavior, group identities, group history, self/non-self differentiation competences, communicative competences,* etc.*). Then, the empirical results of molecular biology would mean that RNA sociology could serve as empirical non-mechanistic explanation of social interactions: from single RNA stem loops to group (identity) building, self/non-self identification competence, the emergence of context-dependent interactions, and the cooperation between RNA groups and their host organisms, most of them counterbalanced by addiction modules. Additionally, RNA sociology could explain the *de novo* generation of nucleic acid sequences and their coherent integration into pre-existing ones, innovation by variations in RNA stem loops and, last but not least, innovative genetic identity by co-evolution.

## References

[1] Witzany G. (2010). Biocommunication and Natural Genome Editing.

[2] Witzany G. (2014). Pragmatic turn in biology: From biological molecules to genetic content operators. World J. Biol. Chem..

[3] Witzany G. (2011). Biocommunication in Soil Microorganisms.

[4] Witzany G., Baluska F. (2012). Biocommunication of Plants.

[5] Witzany G. (2012). Biocommunication of Fungi.

[6] Witzany G. (2014). Biocommunication of Animals.

[7] Witzany G., Baluska F. (2012). Life’s code script does not code itself. The machine metaphor for living organisms is outdated. EMBO Rep..

[8] Brookfield J.F.Y. (2005). The ecology of the genome. Mobile DNA elements and their hosts. Nat. Rev. Genet..

[9] Mauricio R. (2005). Can ecology help genomics: The genome as ecosystem?. Genetica.

[10] Le Rouzic A., Dupas S., Capy P. (2007). Genome ecosystem and transposable elements species. Gene.

[11] Vennera S., Feschotte C., Biémonta C. (2009). Transposable elements dynamics: Toward a community ecology of the genome. Trends Genet..

[12] Witzany G., Kolb V. (2014). Language and Communication as Universal Requirements for Life. Astrobiology: An Evolutionary Approach.

[13] Shapiro J.A. (2013). How life changes itself: The Read-Write (RW) genome. Phys. Life Rev..

[14] Shapiro J.A. (2014). Constraint and opportunity in genome innovation. RNA Biol..

[15] Villarreal L.P., Witzany G. (2013). The DNA Habitat and its RNA Inhabitants: At the Dawn of RNA Sociology. Genomics Insights.

[16] Witzany G. (2011). The agents of natural genome editing. J. Mol. Cell Biol..

[17] Przybilski R., Hammann C. (2007). The tolerance to exchanges of the Watson-Crick base pair in the hammerhead ribozyme core is determined by surrounding elements. RNA.

[18] Lambowitz A.M., Zimmerly S. (2010). Group II Introns: Mobile Ribozymes that Invade DNA. Cold Spring Harb. Perspect. Biol..

[19] Villarreal L.P., Witzany G. (2013). Rethinking quasispecies theory: From fittest type to cooperative consortia. World J. Biol. Chem..

[20] Smit S., Yarus M., Knight R. (2006). Natural selection is not required to explain universal compositional patterns in rRNA secondary structure categories. RNA.

[21] Higgs P.G., Lehman N. (2014). The RNA world: molecular cooperation at the origins of life. Nat. Rev. Genet..

[22] Gwiazda S., Salomon K., Appel B., Müller S. (2012). RNA self-ligation: From oligonucleotides to full length ribozymes. Biochimie.

[23] Müller S., Appel B., Krellenberg T., Petkovic S. (2012). The Many Faces of the Hairpin Ribozyme: Structural and Functional Variants of a Small Catalytic RNA. IUBMB Life.

[24] Villarreal L.P., Witzany G. (2012). The Addiction Module as a Social Force. Viruses: Essential Agents of Life.

[25] Kobayashi I. (2001). Behavior of restriction-modification systems as selfish mobile elements and their impact on genome evolution. Nucleic Acids Res..

[26] Mruk I., Kobayashi I. (2014). To be or not to be: Regulation of restriction-modification systems and other toxin-antitoxin systems. Nucleic Acids Res..

[27] Villarreal L.P. (2011). Viral Ancestors of Antiviral Systems. Viruses.

[28] Villarreal L.P., Lopez-Larrea C. (2012). Viruses and Host Evolution: Virus Mediated Self Identity. Self and Nonself.

[29] Nieva J.L., Madan V., Carrasco L. (2012). Viroporins: Structure and biological functions. Nat. Rev. Microbiol..

[30] Gerdes K., Wagner E.G. (2007). RNA antitoxins. Curr. Opin. Microbiol..

[31] Villarreal L.P. (2009). Origin of Group Identity: Viruses, Addiction and Cooperation.

[32] Lincoln T.A., Joyce G.F. (2009). Self-sustained replication of an RNA enzyme. Science.

[33] Stoddard B.L. (2011). Homing endonucleases: From microbial genetic invaders to reagents for targeted DNA modification. Structure.

[34] Ohmori R., Saito H., Ikawa Y., Fujita Y., Inoue T. (2011). Self-replication reactions dependent on tertiary interaction motifs in an RNA ligase ribozyme. J. Mol. Evol..

[35] Witzany G. (2009). Noncoding RNAs: Persistent viral agents as modular tools for cellular needs. Ann. N. Y. Acad. Sci..

[36] Steitz J., Borah S., Cazalla D., Fok V., Lytle R., Mitton-Fry R., Riley K., Samji T. (2011). Noncoding RNPs of Viral Origin. Cold Spring Harb. Perspect. Biol..

[37] Witzany G. (2008). The viral origins of telomeres, telomerases and their important role in eukaryogenesis and genome maintenance. Biosemiotics.

[38] Alliegro M.C. (2011). The centrosome and spindle as a ribonucleoprotein complex. Chromosome Res..

[39] Alliegro M.A., Henry J.J., Alliegro M.C. (2010). Rediscovery of the nucleolinus, a dynamic RNA-rich organelle associated with the nucleolus, spindle, and centrosomes. Proc. Natl. Acad. Sci. USA.

[40] Chan C.W., Chetnani B., Mondragon A. (2013). Structure and function of the T-loop structural motif in noncoding RNAs. Wiley Interdiscip. Rev. RNA.

[41] Staple D.W., Butcher S.E. (2005). Pseudoknots: RNA Structures with Diverse Functions. PLoS Biol..

[42] Brierley I., Gilbert R.J.C., Pennell S. (2008). RNA pseudoknots and the regulation of protein synthesis. Biochem. Soc. Trans..

[43] Briones C., Stich M., Manrubia S.C. (2009). The dawn of the RNA World: Toward functional complexity through ligation of random RNA oligomers. RNA.

[44] Peselis A., Serganov A. (2014). Structure and function of pseudoknots involved in gene expression control. Wiley Interdiscip. Rev. RNA.

[45] Thapar R., Denmon A.P., Nikonowicz E.P. (2013). Recognition modes of RNA tetraloops and tetraloop-like motifs by RNA-binding proteins. Wiley Interdiscip. Rev. RNA.

[46] Dixon R.J., Eperon I.A., Samani N.J. (2007). Complementary intron sequence motifs associated with human exon repetition: A role for intragenic, inter-transcript interactions in gene expression. Bioinformatics.

[47] Tomizawa J., Gesteland R.F., Cech T.R., Atkins J.F. (1993). Evolution of Functional Structures of RNA. The RNA World.

[48] Forsdyke D.R. (1995). A Stem-Loop“Kissing” Model for the Initiation of Recombination and the Origin of Introns. Mol. Biol. Evol..

[49] Mattick J.S. (2009). Deconstructing the dogma: a new view of the evolution and genetic programming of complex organisms. Ann. N. Y. Acad. Sci..

[50] Malone C.D., Hannon G.J. (2009). Small RNAs as Guardians of the Genome. Cell.

[51] Ng S.Y., Stanton L.W. (2013). Long non-coding RNAs in stem cell pluripotency. Wiley Interdiscip. Rev. RNA.

[52] Amaral P.P., Dinger M.E., Mercer T.R., Mattick J.S. (2008). The eukaryotic genome as an RNA machine. Science.

[53] Farazi T.A., Juranek S.A., Tuschl T. (2008). The growing catalog of small RNAs and their association with distinct Argonaute/Piwi family members. Development.

[54] Filipowicz W. (2000). Imprinted expression of small nucleolar RNAs in brain: Time for RNomics. Proc. Natl. Acad. Sci. USA.

[55] Taft R.J., Glazov E.A., Lassmann T., Hayashizaki Y., Carninci P., Mattick J.S. (2009). Small RNAs derived from snoRNAs. RNA.

[56] Weber M.J. (2006). Mammalian Small Nucleolar RNAs Are Mobile Genetic Elements. PLoS Genet..

[57] Dieci G., Preti M., Montanini B. (2009). Eukaryotic snoRNAs: A paradigm for gene expression flexibility. Genomics.

[58] Doench J.G., Petersen C.P., Sharp P.A. (2003). siRNAs can function as miRNAs. Genes Dev..

[59] Bartel D.P. (2004). MicroRNAs: Genomics, biogenesis, mechanism and function. Cell.

[60] Bartel D.P. (2008). MicroRNAs: target recognition and regulatory functions. Cell.

[61] Fire A. (2005). Nucleic acid structure and intracellular immunity: Some recent ideas from the world of RNAi. Q. Rev. Biophys..

[62] Sontheimer E.J., Carthew R.W. (2005). Silence from within: endogenous siRNAs and miRNAs. Cell.

[63] Obbard D.J., Gordon K.H., Buck A.H., Jiggins F.M. (2009). The evolution of RNAi as a defence against viruses and transposable elements. Philos. Trans. R. Soc. Lond. B Biol. Sci..

[64] Piriyapongsa J., Jordan I.K. (2008). Dual coding of siRNAs and miRNAs by plant transposable elements. RNA.

[65] Hunter P. (2008). The great leap forward. Major evolutionary jumps might be caused by changes in gene regulation rather than the emergence of new genes. EMBO Rep..

[66] Larson B.C., Jensen R.P., Lehman N. (2012). The Chemical Origin of Behavior is Rooted in Abiogenesis. Life.

[67] Eigen M. (1971). Selforganization of matter and the evolution of biological macromolecules. Naturwissenschaften.

[68] Gott J.M. (2003). Expanding genome capacity via RNA editing. Comptes Rendus Biol..

[69] Takenaka M., Verbitskiy D., van der Merwe J.A., Zehrmann A., Brennicke A. (2008). The process of RNA editing in plant mitochondria. Mitochondrion.

[70] Smith H.C., Goeringer H.U. (2008). Editing Informational Content of Expressed DNA Sequences and Their Transcripts. RNA Editing.

[71] Grosjean H., Bjork G.R. (2004). Enzymatic conversion of cytidine to lysidine in anticodon of bacterial isoleucyl-tRNA—an alternative way of RNA editing. Trends Biochem. Sci..

[72] Alfonzo J.D., Goeringer H.U. (2008). Editing of tRNA for Structure and Function. RNA Editing.

[73] Homann M., Goeringer H.U. (2008). Editing Reactions from the Perspective of RNA Structure. RNA Editing.

[74] Jantsch M.F., Oehmann M., Goeringer H.U. (2008). RNA Editing by Adenosine Deaminases that Act on RNA (ADARs). RNA Editing.

[75] Hesselberth J.R. (2013). Lives that introns lead after splicing. Wiley Interdiscip. Rev. RNA.

[76] Carnes J., Stuart K., Goeringer H.U. (2008). Working Together: The RNA Editing Machinery in Trypanosoma brucei. RNA Editing.

[77] House A.E., Lynch K.W. (2008). Regulation of alternative splicing: More than just the ABCs. J. Biol. Chem..

[78] Matlin A.J., Moore M.J. (2007). Spliceosome assembly and composition. Adv. Exp. Med. Biol..

[79] Matera A.G., Wang Z. (2014). A day in the life of the spliceosome. Nat. Rev. Mol. Cell Biol..

[80] Belfort M., Weiner A. (1997). Another Bridge between Kingdoms: tRNA Splicing in Archaea and Eukaryotes. Cell.

[81] Pyle A.M., Lambowitz A.M., Gesteland R.F., Cech T.R., Atkins J.F. (2006). Group II Introns: Ribozymes that Splice RNA and Invade DNA. The RNA World.

[82] Forterre P., Prangishvili D. (2009). The great billion-year war between ribosome- and capsid-encoding organisms (cells and viruses) as the major source of evolutionary novelties. Ann. N. Y. Acad. Sci..

[83] Turowksi T.W., Tollervey D. (2014). Cotranscriptional events in eukaryotic ribosome synthesis. Wiley Interdiscip. Rev. RNA.

[84] Hernandez-Verdun D. (2011). Assembly and disassembly of the nucleolus during the cell cycle. Nucleus.

[85] Belousoff M.J., Davidovich C., Zimmerman E., Caspi Y., Wekselman I., Rozenszajn L., Shapira T., Sade-Falk O., Taha L., Bashan A. (2010). Ancient machinery embedded in the contemporary ribosome. Biochem. Soc. Trans..

[86] Moore P.B., Steitz T.A. (2011). The Roles of RNA in the Synthesis of Protein. Cold Spring Harb. Perspect. Biol..

[87] Hamann C., Westhof E. (2007). Searching genomes for ribozymes and riboswitches. Genome Biol..

[88] Harish A., Caetano-Anolles G. (2012). Ribosomal History Reveals Origins of Modern Protein Synthesis. PLoS One.

[89] Lang B.F., Jakubkova M., Hegedusova E., Daoud R., Forget L., Brejova B., Vinar T., Kosa P., Fricova D., Nebohacova M. (2014). Massive programmed translational jumping in mitochondria. Proc. Natl. Acad. Sci. USA.

[90] Todd G.C., Walter N.G. (2013). Secondary structure of bacteriophage T4 gene 60 mRNA: Implications for translational bypassing. RNA.

[91] Daoud R., Forget L., Lang B.F. (2012). Yeast mitochondrial RNase P, RNase Z and the RNA degradosome are part of a stable supercomplex. Nucleic Acids Res..

[92] Maizels A., Weiner A.M., Gesteland R.F., Cech T.R., Atkins J.F. (1993). The Genomic Tag Hypothesis: Modern Viruses as Molecular Fossils of Ancient Strategies for Genomic Replication. The RNA World.

[93] Maizels N., Weiner A.M., Yue D., Shi P.Y. (1999). New evidence for the genomic tag hypothesis: Archaeal CCA-adding enzymes and tRNA substrates. Biol. Bull..

[94] Randau L., Calvin K., Hall M., Yuan J., Podar M., Li H., Söll D. (2005). The heteromeric Nanoarchaeum equitans splicing endonuclease cleaves noncanonical bulge-helix-bulge motifs of joined tRNA halves. Proc. Natl. Acad. Sci. USA.

[95] Xiong Y., Steitz T.A. (2004). Mechanism of transfer RNA maturation by CCA—adding enzyme without using an oligonucleotide template. Nature.

[96] Wolf Y.I., Koonin E.V. (2007). On the origin of the translation system and the genetic code in the RNA world by means of natural selection, exaptation, and subfunctionalization. Biol. Direct.

[97] Sun F.J., Caetano-Anollés G. (2008). Evolutionary patterns in the sequence and structure of transfer RNA: Early origins of Archaea and viruses. PLoS Comput. Biol..

[98] Phizicky E.M. (2005). Have tRNA, will travel. Proc. Natl. Acad. Sci. USA.

[99] Wegrzyn G., Wegrzyn A. (2008). Is tRNA only a translation factor or also a regulator of other processes?. J. Appl. Genet..

[100] Randau L., Söll D. (2008). Transfer RNA genes in pieces. EMBO Rep..

[101] Maute R.L., Dalla-Favera R., Basso K. (2013). RNAs with multiple personalities. Wiley Interdiscip. Rev. RNA.

[102] Huda A., Mariño-Ramírez L., Jordan I.K. (2010). Epigenetic histone modifications of human transposable elements: Genome defense* versus* exaptation. Mob. DNA.

[103] Mattick J.S. (2010). The central role of RNA in the genetic programming of complex organisms. Anais da Academia Brasileira de Ciências.

[104] Mattick J.S., Taft R.J., Faulkner G.J. (2010). A global view of genomic information—moving beyond the gene and the master regulator. Trends Genet..

[105] Mercer T.R., Mattick J.S. (2013). Understanding the regulatory and transcriptional complexity of the genome through structure. Genome Res..

[106] Qureshi I.A., Mehler M.F. (2012). Emerging roles of non-coding RNAs in brain evolution, development, plasticity and disease. Nat. Rev. Neurosci..

[107] Barry G., Mattick J. (2012). The role of regulatory RNA in cognitive evolution. Trends Cogn. Sci..

[108] Blaze J., Roth T.L. (2012). Epigenetic mechanisms in learning and memory. Wiley Interdiscip. Rev. Cogn. Sci..

[109] Slotkin R.K., Martienssen R. (2007). Transposable elements and the epigenetic regulation of the genome. Nat. Rev. Genet..

[110] Matera A.G., Terns R.M., Terns M.P. (2007). Non-coding RNAs: Lessons from the small nuclear and small nucleolar RNAs. Nat. Rev. Mol. Cell Biol..

[111] Mitchell R.S., Beitzel B.F., Schroeder A.R.W., Shinn P., Chen H., Berry C.C., Ecker J.R., Bushman F.D. (2004). Retroviral DNA Integration: ASLV, HIV, and MLV Show Distinct Target Site Preferences. PLoS Biol..

[112] Diemer G.S., Stedman K.M. (2012). A novel virus genome discovered in an extreme environment suggests recombination between unrelated groups of RNA and DNA viruses. Biol. Direct.

[113] Villarreal L.P. (2005). Viruses and the Evolution of Life.

[114] Ryan F. (2009). Virolution.

[115] Witzany G. (2012). Viruses: Essential Agents of Life.

[116] Villarreal L.P. (2009). The Source of Self. Genetic Parasites and the Origin of Adaptive Immunity. Ann. N. Y. Acad. Sci..

[117] Witzany G., Witzany G. (2012). From Molecular Entities to Competent Agents: Viral Infection-Derived Consortia Act as Natural Genetic Engineers. Viruses: Essential Agents of Life.

[118] Geuking M.B., Weber J., Dewannieux M., Gorelik E., Heidmann T., Hengartner H., Zinkernagel R.M., Hangartner L. (2009). Recombination of Retrotransposon and Exogenous RNA Virus Results in Nonretroviral cDNA Integration. Science.

[119] Gao X., Havecker E.R., Baranov P.V., Atkins J.F., Voytas D.F. (2003). Translational recoding signals between gag and pol in diverse LTR retrotransposons. RNA.

[120] Pathak K.B., Nagy P.D. (2009). Defective Interfering RNAs: Foes of Viruses and Friends of Virologists. Viruses.

[121] Klenerman P., Hengartner H., Zinkernagel R.M. (1997). A non-retroviral RNA virus persists in DNA form. Nature.

[122] Stoddard B., Belfort M. (2010). Social networking between mobile introns and their host genes. Mol. Microbiol..

[123] Villarreal L.P., Witzany G. (2010). Viruses are essential agents within the roots and stem of the tree of life. J. Theor. Biol..

